# Effect of a dual‐boarded emergency–psychiatry physician on psychiatric consultations in the emergency department: A pre–post study in Japan

**DOI:** 10.1002/pcn5.70291

**Published:** 2026-01-23

**Authors:** Takero Terayama, Shogo Takeshita, Nobuhisa Kobayashi, Hiroaki Toda, Takehito Sawamura

**Affiliations:** ^1^ Department of Emergency Medicine Self‐Defense Forces Central Hospital Setagaya Tokyo Japan; ^2^ Department of Psychiatry, School of Medicine National Defense Medical College Tokorozawa Saitama Japan; ^3^ Department of Psychiatry Self‐Defense Forces Central Hospital Setagaya Tokyo Japan

**Keywords:** dual‐boarded physicians, emergency medical services, emergency psychiatry, liaison psychiatry, psychiatric consultation

## Abstract

**Aim:**

To evaluate the impact of assigning a dual‐boarded emergency–psychiatry physician to the emergency department (ED) on the frequency, timing, and nature of psychiatric consultations. Access to psychiatric evaluations in EDs is often limited, particularly in Japan, where general hospitals with psychiatric services are declining. Integrating dual‐boarded physicians in emergency medicine and psychiatry may address unmet psychiatric needs.

**Methods:**

This retrospective, single‐center, pre–post study was conducted at a secondary emergency hospital in Tokyo, Japan. All patients transported by ambulance between October 2020 and September 2024 were included in this study. An a priori sample size calculation was not performed; instead, the study size was determined by all consecutive eligible patients during this predefined observation period. The intervention involved the continuous assignment of a dual‐boarded physician in October 2022. The primary outcome was the number of psychiatric consultations initiated by emergency physicians. Secondary outcomes included reasons for consultation, patient characteristics, physiological indicators at ED presentation, emergency procedures at admission, consultation setting, time from admission to consultation, and clinical outcomes; for patients with multiple consultations during the same hospitalization, only the initial consultation was analyzed.

**Results:**

Among 23,916 patients, 137 psychiatric consultations were analyzed. Consultations increased 3.41‐fold post‐intervention (31 vs. 106), with the consultation rate increasing from 0.29% to 0.81%. High‐risk psychiatric presentations, including suicidal ideation (40.6% vs. 12.9%, *p* = 0.005), self‐harm (42.5% vs. 19.4%, *p* = 0.019), and overdose (33.0% vs. 12.9%, *p* = 0.040), were significantly more common post‐intervention, whereas delirium was less frequent (2.8% vs. 25.8%, *p* < 0.001). Vital signs at ED presentation and emergency procedures at admission were comparable between groups. Time from admission to consultation was shorter post‐intervention (median 1 vs. 3 days, *p* = 0.007). The median hospital stay was also shorter from 22 to 8 days (*p* = 0.001). There was no significant change in the psychiatric hospital transfer rates.

**Conclusion:**

This study demonstrated that the 2‐year assignment of one dual‐boarded emergency–psychiatry physician was associated with an increased acceptance of more complex psychiatric cases in the ED, particularly those involving self‐harm and suicidal ideation.

## INTRODUCTION

Emergency departments (EDs) increasingly serve as frontlines for individuals experiencing acute psychiatric crises. In the United States, mental health–related visits now account for approximately 7%–10% of all ED encounters. From 2006 to 2014, psychiatric presentations rose by 40%, and incidents involving suicidal ideation or attempts increased more than fourfold.^1^, ^2^ However, access to psychiatric specialists remains severely constrained, with patients commonly experiencing delays exceeding 7 h and frequently being admitted to non‐psychiatric wards while awaiting evaluation.^3^ These prolonged delays—termed psychiatric “boarding”—substantially extend ED length of stay, with psychiatric patients spending on average, three times longer in the ED than those without psychiatric conditions.

In Japan, access to emergency psychiatric care has been hampered by the continued decline of general hospitals offering psychiatric services. Psychiatry, often deemed financially unviable, has been progressively eliminated from the general hospital system. A recent position paper by the Japanese Society of General Hospital Psychiatry reported a marked decline in the number of hospitals with psychiatric departments, restricting the availability of specialist consultations in the EDs. According to the same report, only 41% of secondary medical areas were covered by general hospitals with psychiatric inpatient beds, and only 20 of Japan's 47 prefectures exceeded 50% coverage. Thus, emergency physicians in many regions must independently manage psychiatric emergencies amid rising demand. National surveys further documented an 8% reduction in psychiatric beds and a 9% decline in psychiatric facilities between 2002 and 2007, reflecting a persistent nationwide contraction in the psychiatric infrastructure.^4^

Ideally, optimizing emergency psychiatric care requires coordination of emergency and psychiatric resources at a regional level, including timely access to specialist evaluation, inpatient capacity, and interfacility transfer pathways. However, establishing such regional systems often demands substantial inter‐institutional coordination, administrative preparation, and sustained workforce availability, which may be difficult to achieve in many settings. Under these constraints, deploying a dual‐boarded emergency–psychiatry physician may represent a pragmatic, low‐barrier strategy that can provide an immediate clinical bridge between emergency and psychiatric care, particularly during the initial “startup” phase before broader regional integration becomes feasible. In response, Japan's Ministry of Health, Labour, and Welfare has urged enhanced collaboration between mental health and general medical services, emphasizing the need for integrated care for patients with mood disorders, developmental disorders, dementia, and concurrent physical illnesses. Under these circumstances, the continuous presence of dual‐boarded physicians trained in both emergency medicine and psychiatry within the ED may offer a viable solution to ensure uninterrupted holistic care for patients presenting with complex needs. Notably, the recent introduction of the Emergency Behavioral Health (EBH) Focused Practice Designation by the American Board of Emergency Medicine (ABEM) in 2025 underscores the international recognition of the need to enhance psychiatric competencies among emergency physicians.^5^

Nevertheless, empirical evidence evaluating the effects of these specialists on the frequency and nature of psychiatric consultations remains scarce. We hypothesized that the continuous assignment of a dual‐boarded emergency–psychiatry physician in the ED would facilitate the acceptance of patients with psychiatric problems and increase the number of patients with more severe psychiatric conditions. To address this gap, we conducted a single‐center, pre–post comparative study to examine the effects of deploying dual‐boarded physicians in an ED. Importantly, beyond describing a staffing model within a single institution, evaluating this approach may inform how hospitals can initiate more integrated emergency psychiatric care with minimal structural change and may serve as an implementation‐relevant first step toward broader system‐level collaboration.

## METHODS

### Study design

This single‐center, retrospective, pre–post study was conducted at the Self‐Defense Forces Central Hospital (SDFCH) and was approved by the institutional ethics committee (Approval ID: 06‐007). We limited the observation period to 2 years before and after the assignment of the dual‐boarded physician. Beyond this timeframe, the hospital adopted a revised institutional policy to accept a higher proportion of critically ill patients.

Because changes in ED operational policy could substantially affect patient case mix and consultation patterns, extending the study period was considered inappropriate for isolating the effect of the exposure. T.T., a physician board‐certified in both emergency medicine and psychiatry, conducted all stages of the study, including data collection, data extraction, outcome assessment, and statistical analyses. Variables were abstracted using predefined operational definitions, and consultation cases were identified from structured consultation order data to minimize misclassification.

### Participants

We included all patients transported by ambulance to the SDFCH ED between October 1, 2020, and September 30, 2024. For cases in which multiple consultations occurred during the same hospitalization, only the initial consultation was included in the analysis.

### Emergency department system at SDFCH

SDFCH is a secondary emergency care facility located adjacent to central Shibuya. While designated as the final referral hospital for the Japan Self‐Defense Forces, the hospital provides emergency care to the general public at a level comparable to civilian hospitals. It operates a 5‐bed emergency room (ER) and a 300‐bed inpatient service that includes a 5‐bed intensive care unit. The hospital receives approximately 6000 ambulances and 12,000 ED visits annually. The ED is staffed 24 h a day by board‐certified emergency physicians. Each shift typically included one emergency physician, one resident, and two nurses.

### Psychiatric services at SDFCH

Although SDFCH employs full‐time psychiatrists, it does not have dedicated psychiatric inpatient beds. The psychiatric team primarily functions as a liaison service and responds to consultation requests from other departments. Outside regular working hours, psychiatrists are available on an on‐call basis; however, in practice, they are rarely called to provide in‐person assessments or telephone consultations. Requests for urgent psychiatric evaluation—such as those prompted by imminent suicidal ideation—occur only once or twice per year. Consequently, most after‐hours psychiatric presentations are managed by emergency physicians within the scope of their clinical practice.

### Exposure

Exposure was defined as the assignment of a single emergency physician, dual board‐certified in psychiatry and emergency medicine, to the ED during the latter half of the study period (October 2022 to September 2024). He was a mid‐career physician with 15 years of postgraduate experience and 10 years of tertiary emergency care experience. He currently serves as the chief of the ED, oversees clinical operations, leads educational activities for residents and fellows, conducts weekly inpatient conferences, and maintains close communication with the ward nursing leadership. In addition, he is appointed to an institutional medical safety role and serves on a hospital committee that systematically reviews in‐hospital incidents, including episodes of violence and other critical conflicts.

### Collaboration with the psychiatry department

He worked in close collaboration with in‐house psychiatrists. For non‐urgent cases, psychiatric evaluations were deferred to daytime. He independently assessed patients requiring urgent psychiatric intervention, particularly those presenting with concurrent somatic and psychiatric symptoms.

### Specialist certification in psychiatry and emergency medicine in Japan

In Japan, physicians can independently obtain board certifications for psychiatry and emergency medicine. After completing a 2‐year postgraduate rotation program, physicians underwent several years of specialty training. Psychiatric board certification, conferred by the Japanese Society of Psychiatry and Neurology, requires completion of an accredited residency and successful completion of a national examination. Emergency medicine certification, governed by the Japanese Association for Acute Medicine, requires a 3‐year training program, successful completion of a written examination, and fulfillment of clinical case requirements. The minimum time required to obtain both certifications sequentially is approximately 8–10 years after graduation.

### Primary outcome

The primary outcome was a change in the number of psychiatric consultations initiated by emergency physicians. Consultations were identified using electronic order data extracted from the hospital warehouse.

### Secondary outcomes

Secondary outcomes are as follows: (1) changes in patient characteristics, physiological indicators at ED, and clinical outcomes among those receiving consultations, and (2) differences in consultation content and intent.

### Measurements

Psychiatric history was obtained from admission records and, when available, from primary care physician documentation. Diagnoses were made according to the International Classification of Diseases, 10th Revision (ICD‐10). Diagnoses established after admission were recorded as “unknown.” Patients' social background information was primarily derived from a dedicated admission assessment sheet, which is routinely used to comprehensively collect socioeconomic factors and social support information at the time of hospitalization. Clinical outcomes included length of hospital stay and subsequent psychiatric hospitalization, defined as direct transfer from the ED to a psychiatric facility.

Vital signs at ED presentation (heart rate, systolic blood pressure, diastolic blood pressure, Glasgow Coma Scale, and body temperature) were extracted from ED records. Emergency procedures at admission (intubation, use of any vasopressor, use of any sedative and/or physical restraint, and emergency operation) were identified from procedure/medication records. Socioeconomic background (reliable key person, cohabitant, lack of appropriate social support, and public assistance recipient) and regular psychotropic medications (antidepressants, antipsychotics, mood stabilizers, anxiolytics, antiepileptic drugs, and other psychotropic medications) were abstracted from admission documentation and medication reconciliation records.

The primary diagnosis at admission was categorized as altered mental status, infectious diseases, other internal causes, acute drug poisoning, traumatic injury, temperature‐related disorders, any psychiatric symptoms, and other external causes. Cases initially presenting with altered mental status that were later diagnosed as drug overdose were classified as altered mental status; the category “any psychiatric symptoms” included suicidal ideation; and when categories overlapped, cases were classified under physical reasons, as ED disposition is primarily determined by physical conditions. Consultation setting was classified as ER or inpatient, and time from admission to consultation was defined as the number of days from hospital admission to the first psychiatric consultation order (0 indicates the day of admission).

Reasons for consultation were categorized as follows: delirium, suicidal ideation or attempt, self‐harm, drug overdose (including intentional ingestion of prescription or over‐the‐counter medications), dementia, violence or agitation, evaluation of preexisting psychiatric conditions (including continuation of maintenance therapy), new‐onset psychiatric symptoms, and differential diagnosis of psychiatric disorders (e.g., dissociative states or psychiatric causes of altered consciousness). Multiple categories were permitted for each case.

### Statistical analysis

All analyses were conducted using R version 4.3.0 (R Foundation for Statistical Computing, Vienna, Austria) in the RStudio environment. Continuous variables are presented as medians with interquartile ranges (IQRs), and categorical variables as counts and percentages. Patients treated during the first 2 years (pre‐exposure group) were compared with those treated during the final 2 years (post‐exposure group). Between‐group comparisons were performed using Student's *t*‐test or the Mann–Whitney *U* test for continuous variables and the chi‐square test for categorical variables. The primary outcomes and patient characteristics are summarized in the tables. Missing data were not imputed, and analyses were conducted using available data only.

## RESULTS

Data completeness was high, with no missing data for the primary outcome and minimal missingness (<1%) for key secondary variables.

### Participants and primary outcome

**Figure 1:**
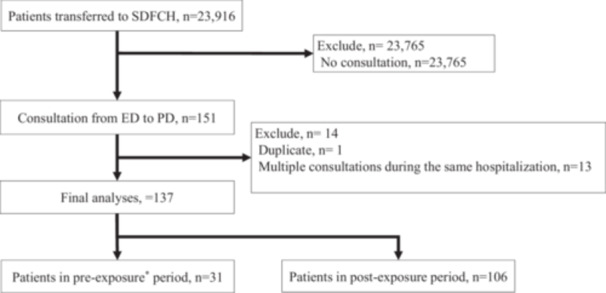
Patient flow chart. ED, emergency department; PD, psychiatry department; SDFCH, Self‐Defense Forces Central Hospital. *Exposure was defined as the presence of a dual‐boarded physician certified in both psychiatry and emergency medicine in the emergency department. During the first 2 years of the study period, no such physician was assigned; in contrast, during the latter 2 years, a dual‐boarded physician engaged in the ED.

Figure 1 illustrates the patient flow. Between October 1, 2020, and September 30, 2024, 23,916 patients were transported to the ED by ambulance. Among these, 151 psychiatric consultations were initiated by emergency physicians. After excluding one duplicate consultation and 13 multiple consultations during the same hospitalization, 137 consultations were included in the final analysis. Of these, 31 occurred during the pre‐exposure period, and 106 occurred during the post‐exposure period, representing a 3.41‐fold increase. The proportion of psychiatric consultations relative to the total ambulance arrivals increased from 0.29% in the pre‐exposure period to 0.81% in the post‐exposure period, indicating a 2.81‐fold increase.

### Secondary outcomes

#### Comparison of patient characteristics between groups

Table 1 summarizes the baseline characteristics. Socioeconomic background and regular psychotropic medications are also summarized. The median age was significantly lower in the post‐exposure group than in the pre‐exposure group (47.5 [26.75–59] vs. 59 [42–78] years, *p* = 0.009). Regular psychotropic medication use was more common in the post‐exposure group, particularly antidepressants, antipsychotics, and anxiolytics. In the pre‐exposure group, the most frequent psychiatric diagnoses were F1 (substance use disorders, 35.5%), F3 (mood disorders, 29.0%), and F0 (organic mental disorders, 22.6%). Conversely, the post‐exposure group was more commonly diagnosed with F3 (35.8%), F2 (schizophrenia, schizotypal, and delusional disorders; 17.0%), and F4 (neurotic, stress‐related, and somatoform disorders; 13.2%) disorders, indicating a shift in the diagnostic distribution. Time from admission to consultation is summarized in Table 3; consultations tended to be initiated earlier in the post‐exposure group. The median length of hospital stay was shorter in the post‐exposure group (8 days [3–16.5 days]) than in the pre‐exposure group (22 [11–35] days, *p* = 0.001). There was no significant difference in the proportion of patients transferred to psychiatric facilities between the two groups (16.1% vs. 14.2%, *p* = 0.776).

**Table 1:** Baseline characteristics and outcomes of the included patients.

	**Pre‐exposure** a **group**	**Post‐exposure group**	***p***
*N*/*N* of emergency transports	31/10,782 (0.29%)	106/13,134 (0.81%)	
Period	2020.10.1–2022.9.30	2022.10.1–2024.9.30	
Sex, male	17 (54.8%)	49 (46.2%)	0.399
Age, years	59 [IQR, 42–78]	47.5 [IQR, 26.75–59]	0.009
Psychiatric past medical histories^b^			
F0	7 (22.6%)	8 (7.5%)	0.018
F1	11 (35.5%)	9 (8.5%)	<0.001
F2	3 (9.7%)	18 (17.0%)	0.406
F3	9 (29.0%)	38 (35.8%)	0.482
F4	4 (12.9%)	14 (13.2%)	1.000
F5	0 (0.0%)	6 (5.7%)	0.337
F6	0 (0.0%)	3 (2.8%)	1
F7	0 (0.0%)	1 (0.9%)	1
F8	1 (3.2%)	2 (1.9%)	0.54
F9	3 (9.7%)	7 (6.6%)	0.694
G^c^	0 (0.0%)	9 (8.5%)	0.21
None	5 (16.1%)	8 (7.5%)	0.152
Socioeconomic background			
Reliable key person	18 (58.1%)	63 (59.4%)	0.891
Cohabitant	19 (61.3%)	51 (48.1%)	0.197
Lack of appropriate social support	20 (64.5%)	55 (51.9%)	0.214
Public assistance recipient	5 (16.1%)	8 (7.5%)	0.152
Regular medications			
Antidepressants	3 (9.7%)	34 (32.1%)	0.012
Antipsychotics	4 (12.9%)	38 (35.8%)	0.015
Mood stabilizers	1 (3.2%)	11 (10.4%)	0.297
Anxiolytics	7 (22.6%)	53 (50.0%)	0.007
Antiepileptic drugs	1 (3.2%)	3 (2.8%)	1.00
Other psychotropic medications	2 (6.5%)	5 (4.7%)	0.656

Abbreviation: IQR, interquartile range.

^a^ Exposure refers to the assignment/presence of a physician board‐certified in both psychiatry and emergency medicine.

^b^ Classification is based on the International Classification of Diseases, 10th Revision (ICD‐10).

^c^ Epilepsy.

#### Comparison of physiological indicators at ER between groups

Among patients who received psychiatric consultation, physiological indicators at ER presentation were comparable between the post‐exposure (*n* = 106) and pre‐exposure (*n* = 31) groups (Table 2). Heart rate (93.5 [78–110] vs. 94 [77–110] bpm, *p* = 0.805), systolic blood pressure (123 [110–139] vs. 127 [110–152] mmHg, *p* = 0.336), diastolic blood pressure (77 [66–85] vs. 82 [60–94] mmHg, *p* = 0.338), Glasgow Coma Scale (15 [13–15] vs. 14 [11–15], *p* = 0.296), and body temperature (36.7 [36.2–37.1]°C vs. 36.6 [36.1–37.1]°C, *p* = 0.472) did not significantly differ between the groups.

**Table 2:** Physiological indicators at emergency room (ER) and clinical outcomes of the included patients.

	**Pre‐exposure** a **group**	**Post‐exposure group**	***p***
Vital signs at ER			
Heart rate, bpm	94 [IQR, 77–110]	93.5 [IQR, 78–110]	0.805
Systolic blood pressure, mmHg	127 [IQR, 110–152]	123 [IQR, 110–139]	0.336
Diastolic blood pressure, mmHg	82 [IQR, 60–94]	77 [IQR, 66–85]	0.338
Glasgow Coma Scale	14 [IQR, 11–15]	15 [IQR, 13–15]	0.296
Body temperature, °C	36.6 [IQR, 36.1–37.1]	36.7 [IQR, 36.2–37.1]	0.472
Emergency procedures at admission			
Intubation	0 (0.0%)	2 (1.9%)	0.441
Use of any vasopressor	0 (0.0%)	2 (1.9%)	0.441
Use of any sedative and/or physical restraint	1 (3.2%)	7 (6.6%)	0.480
Emergency operation	1 (3.2%)	1 (0.9%)	0.403
ER disposition, hospital admission	29 (93.5%)	101 (95.3%)	0.700
Admission due to physical condition	27/29 (93.1%)	90/101 (89.1%)	0.527
Inpatient ward, ICU	2/29 (6.9%)	14/101 (13.9%)	0.524
Primary diagnosis at admission^b^			<0.001
Altered mental status	3 (9.7%)	17 (16.0%)	
Infectious diseases	6 (19.4%)	11 (10.4%)	
Other internal causes	5 (16.1%)	25 (23.6%)	
Acute drug poisoning	0 (0.0%)	26 (24.5%)	
Traumatic injury	9 (29.0%)	12 (11.3%)	
Temperature‐related disorders	3 (9.7%)	0 (0.0%)	
Any psychiatric symptoms	4 (12.9%)	15 (14.2%)	
Other external causes	1 (3.2%)	0 (0.0%)	
Length of hospital stay, days	22 [IQR, 11–35]	8 [IQR, 3–16.5]	0.001
Subsequent psychiatric admission^c^	5 (16.1%)	15 (14.2%)	0.776

Abbreviations: ICU, intensive care unit; IQR, interquartile range.

^a^ Exposure refers to the assignment/presence of a physician board‐certified in both psychiatry and emergency medicine.

^b^ Cases initially presented with altered mental status that were later diagnosed as drug overdose were also classified as altered mental status. The category of any psychiatric symptoms included suicidal ideation. When classification categories overlapped, cases were classified under physical reasons, as emergency department disposition is primarily determined by physical conditions.

^c^ Transfer to a psychiatric hospital at another institution.

#### Comparison of psychiatric consultation content between groups

Table 3 details the clinical setting and reasons for psychiatric consultation. Consultations conducted in the ER tended to be more frequent in the post‐exposure group than in the pre‐exposure group (36 [34.0%] vs. 7 [22.6%]; *p* = 0.23). The proportion of inpatient consultations did not significantly differ between the groups (70 [66.0%] vs. 24 [77.4%], *p* = 0.23). Regarding consultation content, delirium was more frequently identified in the pre‐exposure group (8 [25.8%] vs. 3 [2.8%], *p* < 0.001). Conversely, suicidal ideation (43 [40.6%] vs. 4 [12.9%], *p* = 0.005), self‐harm (45 [42.5%] vs. 6 [19.4%], *p* = 0.019), and drug overdose (35 [33.0%] vs. 4 [12.9%], *p* = 0.04) were significantly more common in the post‐exposure group. No significant differences were observed in the frequency of consultations for dementia (8 [7.5%] vs. 3 [9.7%], *p* = 0.712), evaluation of preexisting psychiatric conditions (30 [28.3%] vs. 10 [32.3%], *p* = 0.67), new‐onset psychiatric symptoms (18 [17%] vs. 5 [16.1%], *p* = 0.911), or differential diagnosis of psychiatric disorders (16 [15.1%] vs. 6 [19.4%], *p* = 0.57). No cases of violence or agitation were reported in either group. Time from admission to consultation tended to be shorter in the post‐exposure group than in the pre‐exposure group (1 day [0–2] vs. 3 days [1–4], *p* = 0.007).

**Table 3:** Comparison of psychiatric consultation content between the groups.

	**Pre‐exposure** a **group**	**Post‐exposure group**	***p***
Consultation setting			
Emergency room	7 (22.6%)	36 (34.0%)	0.23
Inpatient	24 (77.4%)	70 (66.0%)	
Time from admission to consultation, days	3 [IQR, 1–4]	1 [IQR, 0–2]	0.007
Reason or purpose of consultation			
Delirium	8 (25.8%)	3 (2.8%)	<0.001
Suicidal ideation	4 (12.9%)	43 (40.6%)	0.005
Self‐harm	6 (19.4%)	45 (42.5%)	0.019
Overdose of any agents^b^	4 (12.9%)	35 (33.0%)	0.04
Dementia	3 (9.7%)	8 (7.5%)	0.712
Violence or agitation	0 (0%)	0 (0%)	N/A
Evaluation of preexisting psychiatric conditions^c^	10 (32.3%)	30 (28.3%)	0.67
Assessment of new‐onset psychiatric symptoms	5 (16.1%)	18 (17%)	0.911
Differential diagnosis of psychiatric disorder^d^	6 (19.4%)	16 (15.1%)	0.57

Abbreviation: IQR, interquartile range.

^a^ Exposure refers to the assignment/presence of a physician board‐certified in both psychiatry and emergency medicine.

^b^ Most cases involve prescription or over‐the‐counter medications; however, toxic exposures such as carbon monoxide poisoning were also included.

^c^ Includes adjustments to previously prescribed medications during hospitalization.

^d^ Includes differential diagnosis of impaired consciousness, such as stupor and neuroleptic malignant syndrome.

## DISCUSSION

This study demonstrated that the placement of a dual‐boarded physician in psychiatry and emergency medicine was associated with a 3.4‐fold increase in the number of psychiatric consultations. Notably, the consultation content shifted toward a higher proportion of high‐risk psychiatric presentations, including suicidal ideation, self‐harm, and intentional overdose. These findings suggest that even the assignment of a single dual‐boarded emergency–psychiatry physician over a 2‐year period may increase the number of cases requiring psychiatric consultation within the institution and facilitate the identification of patients presenting with more complex psychiatric needs, such as suicidal ideation and self‐harm, in the ED. This study provides novel evidence for the design of integrated emergency psychiatric services.

Although no prior studies have directly examined the effect of dual‐boarded physicians, several investigations have evaluated structural psychiatric interventions in ED settings. A systematic review of liaison psychiatry services found that integrating mental health professionals into ED teams reduced patient wait times, improved staff satisfaction, and enhanced care quality.^6^ In a UK‐based study, extending liaison psychiatry service hours from 40 to 98 h per week resulted in a 13% increase in self‐harm presentations over 3 years, an 11.7% monthly increase in psychosocial assessments, and an 86.1% rise in referrals to external services.^7^ Another report demonstrated that the implementation of psychiatric liaison services significantly increased diagnostic coding of psychiatric disorders within a year.^8^ Collectively, these findings support the premise that enhanced psychiatric presence in emergency care improves access, staff morale, and diagnostic recognition. Existing models of emergency psychiatric care typically require coordinated system development by both psychiatry and emergency medicine departments and often depend on regional collaboration beyond a single institution. In contrast, the deployment of a physician trained in both specialties to the ED represents an approach that can be initiated with substantially fewer structural and organizational preparations.

Our study, which evaluated the effects of a dual‐certified emergency psychiatrist, yielded results broadly consistent with prior evidence. Specifically, we observed an increase in the number and complexity of psychiatric consultations, particularly for high‐risk conditions. These changes likely reflect the establishment of a clinical culture that prioritizes early recognition and proactive psychiatric evaluation, enabled by the presence of physicians with dual expertise. Prior surveys have indicated that emergency physicians often lack training and confidence in psychiatric assessment and may underestimate psychiatric pathology.^9^ Indeed, missed consultations following suicide attempts have occurred at our institution. A dual‐boarded physician may reduce barriers to psychiatric care by serving as an immediately accessible resource. The observed decline in delirium‐related consultations may also reflect the improved management of such cases in emergency medicine itself.

Notably, the recent introduction of the EBH Focused Practice Designation by the ABEM in 2025 reflects the growing recognition of the need to enhance psychiatric competencies among emergency physicians.^5^ In this context, our findings are timely and suggest that staffing models incorporating dual‐boarded clinicians may uncover previously unmet psychiatric needs and influence patient outcomes.

Beyond the ED, physicians with combined training in psychiatry and another primary specialty (e.g., internal medicine–psychiatry or family medicine–psychiatry) have been described as facilitators of integrated care for patients with comorbid medical and psychiatric illness. Surveys suggest that double‐boarded psychiatrists are more likely to practice in consultation–liaison or inpatient settings and to assume leadership roles, potentially serving as bridges between psychiatric and general medical systems.^10^ In addition, graduates of combined family medicine–psychiatry programs report roles spanning both specialties, highlighting how dual training can support integrated‐care delivery.^11^ Moreover, integrated medicine–psychiatry services have been proposed as feasible models that can improve outcomes and reduce costs in medically complex populations.^12^ These lines of evidence support the broader premise that the value of dual training extends beyond emergency psychiatry and may reflect a generalizable advantage of dual‐boarded staffing models.

From an implementation perspective, our findings suggest that embedding a dual‐boarded emergency–psychiatry physician may serve as a pragmatic bridge in general hospitals where 24/7 on‐site psychiatry is difficult to maintain. Rather than replacing psychiatrists, this role may lower the threshold for psychiatric engagement, facilitate earlier risk identification for high‐risk presentations (e.g., suicidality, self‐harm, and overdose), and support multidisciplinary disposition planning for patients with intertwined medical and psychiatric needs.

This study has several limitations. First, the single‐center retrospective pre–post design may limit generalizability to institutions with different psychiatric and emergency care infrastructures. Temporal changes unrelated to the exposure may have confounded the results. In addition, because of the limited number of psychiatric consultations and events, we did not perform multivariable modeling; residual confounding from unmeasured factors likely remains. Second, the analyses were restricted to variables available in the hospital's data warehouse, and long‐term outcomes, such as psychiatric symptom resolution or readmission, could not be assessed. Additionally, because exact timestamps from ED arrival to psychiatric assessment were not consistently available, we could not directly evaluate consultation timing by shift; instead, we reported the interval from hospital admission to consultation as a proxy measure. While such patient‐centered outcomes are ideal, we relied on surrogate indicators such as consultation frequency. Nevertheless, lowering the threshold for psychiatric engagement in the ED remains an important initial step toward improving emergency psychiatric care. Third, exposure involved a single physician. Although the physicians' leadership role may have contributed to the observed effects, standardized workflows and clearly delineated responsibilities were implemented to minimize bias. Finally, all stages of data collection, extraction, and analysis were conducted by a single investigator (T.T.), which may have introduced observer or analytic bias.

This study demonstrated that assigning a physician dual board of psychiatry and emergency medicine to the ED was associated with an increased number of psychiatric consultations, particularly for high‐risk presentations. These findings suggest that staffing can reduce barriers to psychiatric care and promote earlier and more comprehensive interventions for patients with psychiatric comorbidities. Notably, they also indicate that even the assignment of a single dual‐boarded physician may be sufficient to meaningfully transform psychiatric care practices within a single institution, highlighting the potential role of dual‐boarded physicians as a practical and scalable starting point for integrated emergency psychiatric care. Further research with larger sample sizes and multicenter designs across diverse healthcare settings is warranted to validate these findings and inform system‐level implementation strategies. Studies to examine whether dual‐boarded physicians facilitate earlier initiation of psychiatric consultations, deliver more comprehensive psychiatric care in the ED, and ultimately translate these process‐level improvements into better patient outcomes are also required.

## AUTHOR CONTRIBUTIONS

Takero Terayama managed all the procedures for writing the manuscript. Shogo Takeshita, Nobuhisa Kobayashi, Hiroaki Toda, and Takehito Sawamura contributed to the organization and structure of the manuscript.

## CONFLICT OF INTEREST STATEMENT

The authors declare no conflicts of interest.

## ETHICS APPROVAL STATEMENT

This study was conducted at the Self‐Defense Forces Central Hospital (SDFCH) and was approved by the institutional ethics committee (Approval ID: 06‐007).

## PATIENT CONSENT STATEMENT

In accordance with the study design, individual written informed consent was not required. Information regarding the study was made available through a publicly accessible document, and potential participants were informed of their right to opt out.

## CLINICAL TRIAL REGISTRATION

Not applicable.

## Data Availability

The data that support the findings of this study are available on request from the corresponding author. The data are not publicly available due to privacy or ethical restrictions.
